# Reimagining Health Data Exchange: An Application Programming Interface–Enabled Roadmap for India

**DOI:** 10.2196/10725

**Published:** 2018-07-13

**Authors:** Satchit Balsari, Alexander Fortenko, Joaquín A Blaya, Adrian Gropper, Malavika Jayaram, Rahul Matthan, Ram Sahasranam, Mark Shankar, Suptendra N Sarbadhikari, Barbara E Bierer, Kenneth D Mandl, Sanjay Mehendale, Tarun Khanna

**Affiliations:** ^1^ Beth Israel Deaconess Medical Center, Harvard Medical School Department of Emergency Medicine Boston, MA United States; ^2^ Harvard FXB Center for Health and Human Rights Boston, MA United States; ^3^ NewYork-Presyterian Hospital Emergency Medicine New York, NY United States; ^4^ The Human Diagnosis Project Washington DC, DC United States; ^5^ Patient Privacy Rights Boston, MA United States; ^6^ Digital Asia Hub Hong Kong China; ^7^ TriLegal Bangalore India; ^8^ Athenahealth San Francisco, CA United States; ^9^ Harvard Business School Boston, MA United States; ^10^ International Institute of Health Management Research New Delhi India; ^11^ Brigham and Women's Hospital, Harvard Medical School Boston, MA United States; ^12^ Boston Children's Hospital Computational Health Informatics Program Boston, MA United States; ^13^ Harvard Medical School Department of Pediatrics and Department of Biomedical Informatics Boston, MA United States; ^14^ Indian Council of Medical Research New Delhi India

**Keywords:** health information exchange, India, health APIs

## Abstract

In February 2018, the Government of India announced a massive public health insurance scheme extending coverage to 500 million citizens, in effect making it the world’s largest insurance program. To meet this target, the government will rely on technology to effectively scale services, monitor quality, and ensure accountability. While India has seen great strides in informational technology development and outsourcing, cellular phone penetration, cloud computing, and financial technology, the digital health ecosystem is in its nascent stages and has been waiting for a catalyst to seed the system. This National Health Protection Scheme is expected to provide just this impetus for widespread adoption. However, health data in India are mostly not digitized. In the few instances that they are, the data are not standardized, not interoperable, and not readily accessible to clinicians, researchers, or policymakers. While such barriers to easy health information exchange are hardly unique to India, the greenfield nature of India’s digital health infrastructure presents an excellent opportunity to avoid the pitfalls of complex, restrictive, digital health systems that have evolved elsewhere. We propose here a federated, patient-centric, application programming interface (API)–enabled health information ecosystem that leverages India’s near-universal mobile phone penetration, universal availability of unique ID systems, and evolving privacy and data protection laws. It builds on global best practices and promotes the adoption of human-centered design principles, data minimization, and open standard APIs. The recommendations are the result of 18 months of deliberations with multiple stakeholders in India and the United States, including from academia, industry, and government.

## Introduction

### Background

India’s population of over 1.3 billion is served by over 2.5 million health care workers of varying qualifications. The vast majority of clinical interactions are not digitized. In the few instances that they are, the data are not standardized, not interoperable, and not readily accessible to clinicians, researchers, or policy makers [1]. While barriers to easy health information exchange (HIE) are hardly unique to India, the greenfield nature of India’s digital health infrastructure presents an excellent opportunity to avoid the pitfalls of complex, restrictive, digital health systems that have evolved elsewhere.

In February 2018, the Government of India announced a massive public health insurance program under the National Health Protection Scheme (NHPS), offering Indian Rs 500,000 (approximately US $ 7,600) in annual coverage to 100 million households, or nearly 500 million citizens [2]. To meet this bold target, the government will rely on technology to effectively scale services, monitor quality, and ensure accountability. While India has seen great strides in informational technology development and outsourcing, mobile phone penetration, cloud computing, and financial technology, the digital health ecosystem is in its nascent stages and has been waiting for a catalyst to seed the system. The NHPS is expected to provide just this impetus for widespread adoption.

We propose here a federated, patient-centric, application programming interface (API)–enabled health information ecosystem that leverages India’s near-universal mobile phone penetration, universal availability of unique identification (ID) systems, and evolving privacy and data protection laws. The arguments laid out here are the result of an extended set of deliberations that began at an interdisciplinary seminar held at Harvard in September 2016 and have since resulted in potential pathways for prototype development in India.

### The State of Health Data Exchange

Electronic Health Records (EHRs) have traditionally been closed systems, sometimes incapable of sharing access across platforms within the same institution, and almost never across vendors at independent institutions. While more systems now allow patients access to their health-related data, few EHRs give patients control over how their data will move across institutions or be shared between providers. Despite significant legislation, a large portion of health data collected today remains inaccessible due to legitimate concerns over confidentiality and privacy, risk-averse hospital policies, prohibitive costs associated with change, and inertia [3].

While health data have been typically associated with information captured in EHRs, there is growing recognition that data are generated at multiple nodes along the delivery system. For example, at the pharmacist, at the stand-alone imaging facility, at the laboratory, at the general practitioner’s office, at the hospital, at the insurance company, and now, even on one’s wrist [4]. However, the lack of standardization among data storage systems makes it virtually impossible to combine and collate data from multiple sources, resulting in duplication, redundancy, wastage, and delays [5].

The concept of a personal health record (PHR) has long been floated as one potential solution to disjointed health care data [4,6,7]. A PHR relies on a patient-controlled repository where data may be accessed from multiple nodes within the system. Standalone PHRs mostly rely on the patient’s drive and ability to input data [8,9]. Tethered (ie, connected) PHRs are patient-accessible components of electronic medical records linked to an institution or health system [8,9]. Still, there are drawbacks. PHRs seldom allow direct input from or access to entities outside the network. Neither PHR model allows for the development of third-party applications (app) on the patient’s health data repository. Although there is interest from the consumer, widespread adoption of both has been hindered by concerns about data ownership, interoperability, security, and scalability [10-12]. The Ministry of Health and Family Welfare (MoHFW) in India has demonstrated an interest in developing a PHR-based system [13].

In recent years, additional individual and population health data have been generated by wellness gadgets (eg, Fitbit), Web-based diagnostic devices (eg, AliveCor), patient-facing apps (eg, Stanford Healthcare), provider-facing apps (eg, Practo), or researcher-facing apps (like Apple’s Research Kit). These new apps and gadgets create additional silos of health data. In fact, of the 260,000 mHealth apps that existed on the last count, 90% were free—their financial viability predicated on their ability to monetize the data they collect [14]. In the United States (US), the 21^st^ Century Cures Act (2016), mandated that “certified” health information technology (IT) products have APIs that allow health information to be accessed, exchanged, and used “without special effort.” Standardization was not mandated, making interoperability difficult to implement [15]. In India, while the mHealth industry is booming and expected to grow exponentially, there are no legal provisions to regulate access to personal health data that flow in and out of these devices and apps, and sometimes across international borders [16].

The call for data integration, universal compatibility, and portability has come from many quarters. There is no shortage of standards, but few are universally applied. There are standards for nomenclature and terminology, structural and semantic standards, and open source technology platforms that promote secure health information exchange [17]. Entrepreneurs and provider networks have responded to this need for data portability, and the potential for monetizing vast amounts of data, by creating their own ecosystems where the patient is not the final arbiter of data flow [18-21].

There is now sufficient recognition that restricting health data access is detrimental to patient care, provider satisfaction, and health care costs [22]. Conversely, access to health data has been shown to benefit and empower patients [23]. Authorized access to the vast troves of accumulated digital data helps accelerate medical research [24]. In March 2016, the US National Institutes of Health in collaboration with the Office of the National Coordinator for Health IT announced the launch of the Precision Medicine Initiative Sync for Science program. Based on existing community standards and specification efforts, this pilot program gave patients easy access to their data and allowed them to donate it to researchers [25] securely. The most significant challenges facing this initiative are individual and collective concern over data security and privacy, and hospitals’ and practitioners’ reluctance to promote the program.

### Health Data in India

Health is a “state” subject in India, managed and funded by state governments, with part-funding from the Center (ie, the federal government in Delhi). Consequently, there is wide variation in quality of care within and among states [26,27]. On average, 70% of health care is delivered through the private sector, which is comprised of state-of-the-art tertiary facilities, nursing homes, polyclinics, general practitioners and a large workforce of health care providers with no medical qualifications [28]. The public health system is robust in population health interventions like vaccine delivery but struggles to provide quality primary care or specialized services at scale [29]. There are also stark differences in health care services in rural and urban India, with the majority of the medical workforce, and tertiary services gravitating to urban India [30].

Conversations about health information exchange in India must acknowledge these realities, as well as the near absence of digital health information in most clinical transactions. The private sector is mostly not digitized except for major diagnostic laboratory and radiology franchises, and some private hospital networks [31,32]. Hospital chains like the Apollo Group and Max Healthcare Group with advanced EHRs have reached Stage 6 of the Healthcare Information and Management Systems Society classification of EHR adoption [33]. However, EHRs are not yet portable across these institutions, and most systems continue to struggle with physician compliance [31,32,34].

In the public sector, data have been collected through various overlapping, regional or national mandates, or dictated by the needs of sponsoring philanthropic foundations [35-37]. The systems, where they exist, are District Health Information System 2 compatible, and mostly comprised of aggregate data. Longitudinal patient records are a recent (and still rare) phenomena. Select public hospitals have managed to digitize some components of the medical record. The governments of Rajasthan, Andhra Pradesh, Maharashtra, and Tamil Nadu have all sponsored EHR implementation to varying extents [38,39]. The Tamil Nadu system, for example, connects over 1,500 primary health centers, 267 secondary care hospitals, and 17 medical colleges. Sustained investment by the state government has been key to the program’s success and scale [39]. The JJ Group of Hospitals in Mumbai, Maharashtra has logged over 20 million patient visits in the past decade, using the Amrita HIS platform [40]. India’s premier public hospital in Delhi, AIIMs, uses a patient scheduling software aimed at reducing wait times. Several other state government sponsored tertiary hospitals use the e-Aushadhi supply chain management system [41,42]. However, these systems lack portability. They are also limited to government-run health facilities precluding residents from accessing data across state lines or from transporting data across public, and private health care facilities.

At the primary care level, community health workers and clinical staff log data in paper-based notebooks, tablets, excel sheets, and a variety of software applications that differ from state to state. The validity of much of these data is questionable [43,44]. Preliminary analysis from a study by our team at a primary care center in one state in India shows that there are over 3,000 discreet fields of data captured in paper-based and electronic forms, contributing data to 70 different databases, the majority of which are never accessed or used for real-time clinical or policy decision making. These repetitive data collection requirements result in large duplication, wastage, and divergence of limited human resources.

Health data are also captured at hundreds of research institutions across the country, in paper files, personal flash drives, hard drives, and sometimes on institutional servers. There is a general consensus among local researchers that much of these data are never analyzed, and they seldom change clinical practice or care delivery [45,46].

Data do not travel across jurisdictions. For example, the Indian Council of Medical Research, India’s leading body for biomedical research has limited access to the data generated across its various collaborating institutions, and almost none to data generated in private institutions. Critical clinical data with significant individual and public health consequence, like information on compliance and antibiotic resistance, are not portable across institutions. Also, the government’s Revised National TB Control Program cannot follow patients or monitor their care once they choose to seek treatment in the private sector. Even if private sector entities were willing to share data, there are no mechanisms to do so. The lack of interoperability of such critical data has a profoundly negative consequences on existing disease surveillance systems [47].

There is growing recognition among the public sector that all new digitization efforts must conform to prescribed standards. The government adopted Systematized Nomenclature of Medicine (SNOMED) and is making it available free of cost to health systems across India [48]. Organizations like HIMSS and the India Health Information Network are other key stakeholders. While the government intends to establish interoperability standards, the question of change management remains unaddressed. Who will bear the cost of these new systems, and of transitioning the older systems? What will be the institutional and individual incentives? How will the system be seeded, populated and sustained? In the United States, the Health Information Technology for Economic and Clinical Health (HITECH) Act provided incentives (and penalties) for Electronic Health Record (EHR) adoption, at the cost of US $ 34 billion in payments to doctors and hospitals to purchase and promote electronic health systems [49]. A 2017 study comparing hospitals that qualified for monetary incentives for implementing “meaningful use” of EHRs to those that did not qualify, attributed 8 percentage points in adoption growth to the incentives provided by HITECH [50]. India can barely afford such expenditures when health spending is at less than 2% of its GDP [51]. What then would be the much-needed catalyst to stimulate widespread digitization?

Until 2017, the lack of a significant insurance player in the market precluded piggybacking EHR adoption on the billing requirements of payers and providers. Deliberations with critical stakeholders in India facilitated by the authors through 2016 and 2017 focused on the three nodes in the system that were most digitized, namely, laboratories, pharmacies and radiology reports. Still, mechanisms for change were not clear. The 2018 NHPS, with its urgent need for a technological backbone, changes all that—it has potential to finally develop this vision for universal HIE in India.

This approach is, however, not without its dangers. The EHRs in the United States have evolved as very effective billing instruments and provided medicolegal safeguards, but basic patient and provider needs like portability and access were an afterthought and required prohibitively expensive retrofitting. Systems work best, and compliance is highest, when the EHRs can be customized to local workflows, and when they can be modified or upgraded with relative ease and at low costs. Hospital mergers in the United States have resulted in near-uniform systems across vastly different enterprises, changes in which require universal consensus across the ecosystem and entail prohibitive fees charged by EHR corporations and additional re-training costs.

While the NHPS may indeed be the much-awaited catalyst for jumpstarting the digital health ecosystem in India, mandating a one-size-fits-all nationwide billing platform will do irreparable and costly damage—costs the Indian health care system cannot afford to bear [52].

## An Application Programming Interface–Enabled Health Information Exchange for India

Between August 2013 and December 2016, India’s MoHFW released a set of recommendations for electronic health records that outlined vital components of a standardized health care information ecosystem, and a common language for the organization of medical terminology and data [43,53]. The Ministry also instituted the National Digital Health Authority meant to “regulate, develop and deploy digital health along the continuum of care across India [54].” In December 2016, the government’s Centre for Health Informatics released a Request for Proposals for the creation of an integrated health information platform (IHIP), where the exchange would be facilitated via a central storage repository. Low budget allocation and limited information on the proposed architecture precluded most IT service companies from bidding. The contract was finally awarded in January 2018, just weeks before the NHPS was announced [55]. The centralized IHIP as envisioned would be monolithic and prescriptive, risking poor user adoption, high physician burnout, and little meaningful access to the vast data it would generate, as seen in large hospital systems in the United States [52,56]. It risks not being able to leverage future IT developments.

The prototype outlined below argues against the use of a centralized repository of health data. Instead, we submit that the way forward must be an API–enabled, blockchain-based information network in which the personal health record underpins a system where free, real-time flow of data is predicated on consent and authorized access. India is uniquely positioned to build this ecosystem armed with a universal identity system, experience with digitization across multiple other industries, and a sophisticated domestic IT workforce.

We describe below the technical and legal basis for the design proposed.

### Federated Architecture

Our proposed model calls for a federated architecture that acknowledges current and future health information flow; for example, between providers and patients, wearables and EHRs, consumers and pharmacies, physicians and laboratories, or institutions and payers. Collating all data in a national repository for 1.3 billion Indians will prove to be prohibitively expensive, redundant, and wasteful. It would also offer a single point of failure where security breaches would result in colossal data compromise. A federated system would allow data to sit at the source and be recalled on demand.

**Figure 1:**
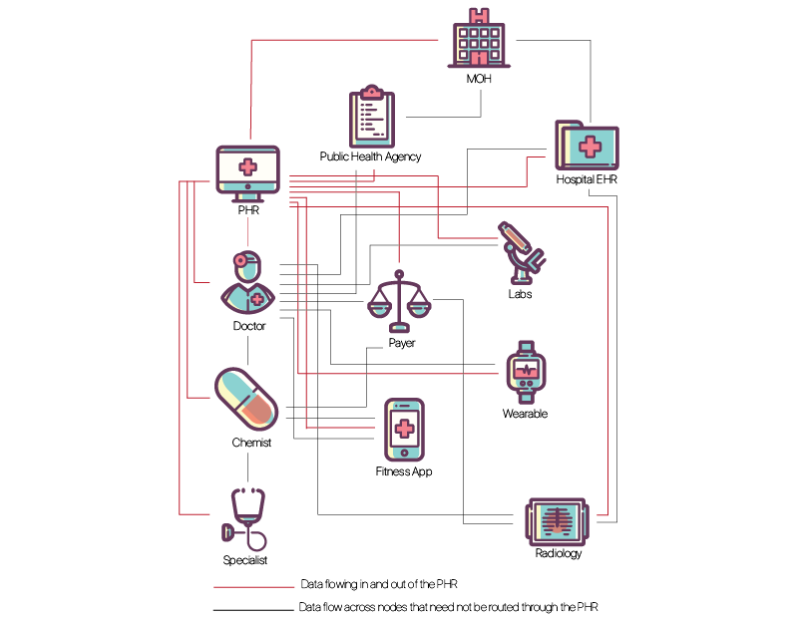
Federated Health Information Exchange Schema: The personal health record (PHR) would access data from existing and novel sources, by preauthorization, waiver or legal mandate. EHR: electronic health record; MOH: Ministry of Health.

An API–enabled federated health data architecture would function on blockchain principles as an “open, distributed ledger that can record transactions between two parties efficiently and in a verifiable and permanent way” [57,58]. Consider a PHR that could query all nodes in the network to receive periodic updates—from wearables, diary entries, pharmacists, doctors, hospitals, diagnostic labs, imaging facilities, and payers. It is possible to map out various permissible pathways through which the data can travel automatically while there may be others through which it cannot pass without the patient’s consent (Figure 1).

**Figure 2:**
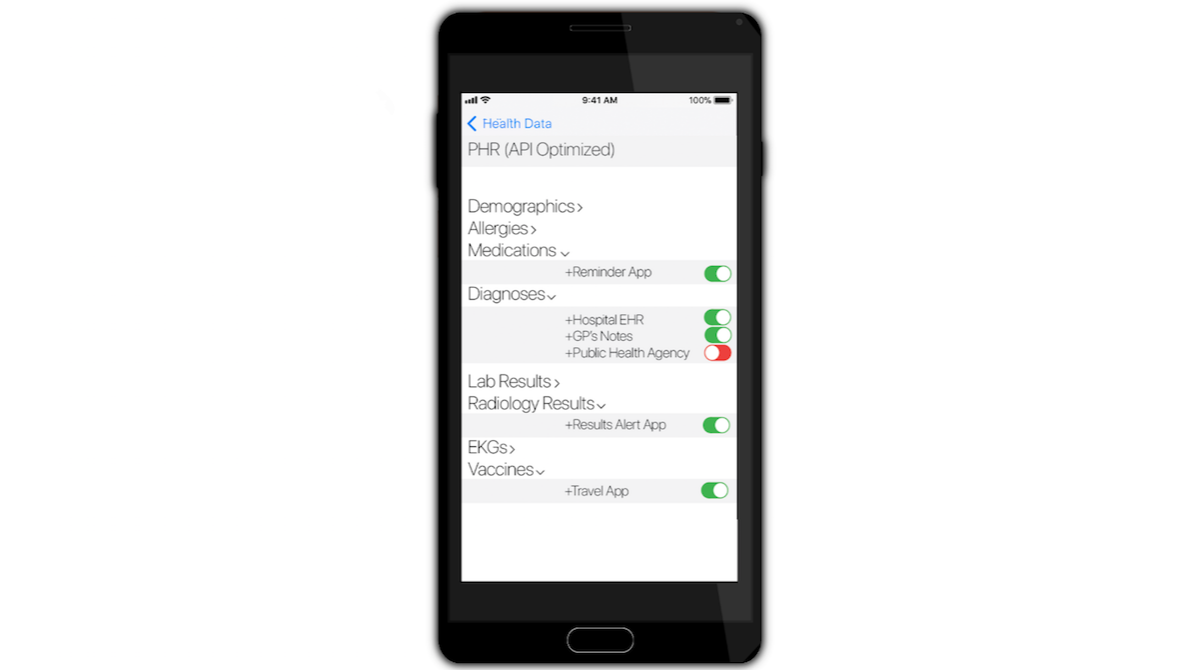
A customizable personal health record (PHR) interface. Through user-driven consent and control, third-party plug-ins (apps) can access the PHR via standard open application programming interfaces (APIs).

An authorized physician—even a virtual “teledoc”—would be able to call for her patient’s entire record, either through pre-authorization, real-time authentication, or waivers in case of emergencies [56]. Diagnostic laboratories should be permitted to send their reports to the patient’s physician who requested the test but will need authorization from the patient to send it to any other doctor (such as one to whom the patient goes for a second opinion). Similarly, a public health agency, duly authorized, should be able to query select de-identified test results across all laboratories in a region of interest, to forecast, monitor, and respond to disease outbreaks [59]. Health system administrators should similarly have access to aggregate data for monitoring delivery, resource utilization and clinical outcomes. Physicians should be able to query their practice patterns. Third-party applications that are built off the patient’s PHR, for example, alerting the patient to vaccine requirement before travel, or triggering reminders based on her medication list, would need the patient’s permission to access data from her PHR (Figure 2).

As long as the user is “recognized” by the system, and therefore has pre-authorization to query particular types of data, access should be easy and near instantaneous. Essentially, the “consent” process is separated in time and place from data flow, allowing timely, secure, exchange of relevant health data between nodes. A federated and distributed network so constructed would obviate the need for constructing large national or regional databases of the patient’s “entire” medical record. Opportunistic synchronization and personal device-based back-up may reasonably mitigate the effect of unreliable electricity and connectivity. Moreover, all these inquiries and interactions should generate audit trails to prevent misuse.

Smartphone penetration in India is expected to reach about 36% of all mobile phone users [60]. While important components of the proposed architecture, mobile phones are not critical. Web-based services, hospital-based kiosks, and patient service facilities can assist those without mobile phones in understanding and accessing their settings. Local regulations should ensure that the default always protects the patient’s interests and privacy.

While seemingly simple in architecture, such a system is predicated on standardization and widespread adoption. We next discuss how there is sufficient precedence and local capacity to favor such sweeping change.

### Universal Unique Patient Identifier

Prima facie, access to the federated architecture would require a universal identifier. All data would be tagged with that unique identifier no matter where the patient interfaced with the medical system. Further, any entity contributing to or extracting from the system would need a unique institution and personal identity tag.

While usually a daunting system to create, the near universal penetration of India’s unique biometric identification program, Aadhaar, offers a solution to this challenge. Aadhaar has been built around the principles of privacy by design, and data minimization, that are particularly relevant in security-sensitive applications like health care. Administered by the Unique Identification Authority of India, the system is actively used today for the central government's direct benefit transfers and subsidies programs and has also been used by several banks and telecom operators. By 2017 over a billion Indian residents were enrolled in Aadhaar, making it the most widely deployed single ID system anywhere in the world.

This astonishing penetration notwithstanding, mandating the linkage of public services to Aadhaar has been problematic, and a subject of constitutional challenges before the Supreme Court, with legitimate concerns of misuse and state overreach [61]. It is possible that the public resistance for using Aadhaar may necessitate the creation of a separate unique medical identity number.

### Local Precedence

The use of APIs would underpin the proposed federated architecture. An API is a set of routines, protocols, and tools built into a software application that enables it to communicate easily with other applications. APIs provide the means to build interoperable software and data exchange services. APIs are already behind the seamless integration from which we benefit every day. They allow Instagram to access the camera on our phones, agnostic to the maker of the device; or allow WhatsApp to access our phone directory (“contact lists”); or a taxi app to use Google maps. Industries like banking, finance, and social media have already successfully tapped into the explosive growth of software applications by adopting API-based solutions [62,63]. Unrelated corporations own most products that communicate via APIs, yet the use of standard APIs powers the entire digital ecosystem.

India’s own experience with wide-scale API adoption in the financial technology (eg, fin-tech) sector has been regarded as hugely successful: The Unified Payments Interface, rolled out in 2016, has demonstrated both the feasibility and the advantages of adopting an API-based ecosystem. Aadhar spurred a range of nationwide API-based IT solutions, collectively referred to as IndiaStack [64]. The Aadhar dashboard eKYC, for example, is a paperless “Know Your Customer” (KYC) process aimed at verifying an individual’s identity. This instant electronic verification has replaced the traditional KYC process that relied on the onerous in person presentation of paper-based copies of official identity documents. The eKYC is currently used across many industries, including banking, utilities, and mobile services, and has logged 4.2 billion transactions in the past four years [65].

The Digital Locker is another successful application that provides a cloud-based storage service to all residents that authorized users can access. Registered Digital Locker organizations can push (or retrieve) electronic copies of documents and certificates (eg, driving license, voter ID, school certificates) directly into the lockers of Indian citizens, once again making credentialing and verification processes near-instantaneous. As of January 2018, nearly 2 billion digital documents have been issued through the Digital Locker API [66]. The widespread adoption of these changes in the financial-tech sector was catalyzed by support from the highest levels of government.

The successful and explosive use of mobile financial services notwithstanding, user-controlled dataflow through the federated network assumes some degree of digital literacy and understanding the ramifications of consent, secondary data use, artificial intelligence algorithms, and so on. Until such time that the Indian populace is assumed to have such knowledge, concomitant local laws will need to require that default data access protects foremost, the patient. We discuss the relevant existing and evolving Indian legal standards in subsequent sections.

### Global Standards

Globally, the health care industry has begun embracing API-based exchange. Open Medical Record System, and platforms like SMART Health IT, developed at Boston Children’s Hospital, have long pursued health data ecosystems anchored in open standard APIs [67,68]. These programs, while successful, had been limited in scale due to the rigid and expensive architecture discussed above. However, the field is rapidly changing. In June 2017, Athenahealth invested US $63 million in a product that improved user-experience and ensured interoperability with other EHRs. The product enabled Athenahealth to open up their APIs for accelerated innovation for their provider base [69]. In January 2018, Apple adopted SMART standards for their Health Kit; others are expected to follow suit. Patients will soon come to expect that their health data be accessed via a range of apps on their phone.

Successful interoperability will rely on widely adopted standardization in data storage and retrieval. Systematized Nomenclature of Medicine-Clinical Terms (SNOMED-CT), Logical Observation Identifiers Names and Codes (LOINC), and RxNorm, a standardizing nomenclature for a medication that can interpret varying vocabulary used by pharmacy and drug interaction software, are increasingly used globally. Health Level 7 (HL7) enables health records and exchanges to be built with typical architecture and structure. Fast Healthcare Interoperability Resources (FHIR), building off HL7 standards, provides data formats and resources for building APIs for facilitating exchange. Project Argonaut, for example, a consortium involving governmental, private and academic health IT leaders, has incorporated these standards to begin work on supporting the uptake of APIs and including them in “meaningful use” regulation [17,70]. India was an early adopter of many of these standards but remains a slow implementer, given the absence of incentives (or penalties) for adoption.

### Data Minimization

The architects of India’s digital health infrastructure, while being compliant with global standards, may consider creating a series of “minimum datasets” for standardization and interoperability. In this article, the use of the term *minimum data set* denotes the least possible number of data points that early digital health information systems in India must include to be useful to a range of stakeholders. Obfuscating digitization with validity, new digitized data collection tools are frequently bloated with information that will either not be used or is already being collected elsewhere. A minimal viable product, instead, will help seed the ecosystem and can be incrementally expanded upon, not unlike the phased requirements seen with HITECH. This approach will allow a vast range of pilots to be tested, evaluated, optimized to meet contextually relevant needs, and then scaled up. Data minimization is also a useful tool in improving security and reducing privacy abuse [71]. In India, focusing on *structured* laboratory data, radiology results, medications lists (ie, in sync with interactions at the chemist), allergies, diagnoses, and essential demographic information would provide enough data to spurn a variety of applications for all involved stakeholders.

### Substitutability

It is imperative that early prototypes pay critical attention to the user experience. Attempts at EHR adoption in India have failed to date due to the untenable combination of very high patient volume and poor usability. Once again, an API–enabled system will allow providers and institutions to select products that are highly customized to the local context and workflow [72]. Such “substitutability” will be central to advancement—apps that access underlying data that can be updated or swapped out for new ones with improved features and usability, just as we currently do with apps from an app store [15].

## The Law

Traditional privacy principles have been articulated in the Organization for Economic Cooperation and Development guidelines first published in the 1980s, revised last in 2013, the European Directive 95/46/EC, and several pieces of national legislation. These principles have come under increasing scrutiny with the power of big data analytics to combine information from discreet datasets. The EU General Data Protection Regulation (GDPR), considered one of the most stringent of data protection laws came into effect on May 25, 2018. It adopts a rights-based framework, placing the individual at the center of the law. In the United States, while the public sector is mostly governed by the Fair Information Practice Principles and related acts, data flow in the private sector is primarily regulated by notice and consent and overseen mostly by the Federal Trade Commission.

As India’s planners imagine its new technologically powered health data ecosystem, hard questions need to be answered. For example, what risks do we pose for individuals and populations by allowing such seamless data travel? Who owns the data? Can such data be sold? If yes, does the patient have a financial claim, even when data are de-identified? What protection measures need to be put in place? What remedy does the patient have? What legal risks do patients, providers, scientists, and governments expose themselves or each other to? Are the technologies for such secure, encrypted, failsafe ecosystems available? What can we learn from other industries? Why have previous attempts failed?

Emerging economies often lack dedicated privacy laws, relying instead on a patchwork of consumer protection laws, telecommunications statutes, human rights provisions and other measures to tackle data breaches, privacy violations, and constitutionally protected rights to equal treatment. However, as government welfare and benefits are increasingly delivered through online platforms on the backs of newly digitized databases, there is a need to ramp up the legal infrastructure in parallel [73]. This need is critically felt when examining the ability of illiterate users to provide informed consent and to exercise control over valuable data. By engineering interoperable systems that default to protect and empower users by offering them control and discretion over data sharing arrangements, one can optimize the benefits of exchange without compromising privacy or security.

Data mining of de-identified information can now reveal very sensitive data [74-76]. Insurance premiums (in a less regulated health care system), for example, can be modified based on zip codes, browser history or seemingly unrelated shopping habits [75,77]. Technology is not neutral, and most systems encode values and biases, however unconscious [78,79]. Evolving research highlights the risks of data-driven or algorithmic decision-making [80,81]. Biometrics, which systems rely upon for identity verification, has been shown to have higher error rates (false positives and false negatives) for darker skin tones [82].

Societal expectations of privacy can also ebb and flow, and the laws need to be nimble enough to accommodate for the fast pace of IT evolution. Only a few years ago, the idea that Google would scan emails to automatically populate a person’s calendar or send alerts was considered highly unacceptable. Today, for many, it is the norm.

In August 2017, the Supreme Court of India ruled that privacy is a fundamental right [83]. The landmark judgment potentially provides the necessary deterrents to data misuse in a health IT ecosystem, in addition to establishing the supremacy of patient’s control over her health data (privacy). The Ministry of Electronics and Information Technology constituted an expert committee to help draft a bill on data protection. The committee released a White Paper on the data protection framework for India, inviting public comment [84]. The White Paper extensively examined best practices elsewhere in the world, with particular attention to European Commission’s GDPR regulations scheduled to go into effect on May 25, 2018.

Responses to the White Paper included recommendations focused on the protection and use of health data, calling for automated but consented flows, easier access, and portability, and without jeopardizing the safety or privacy of vast swathes of India’s digitally illiterate populations. Reviewers opined that health data are generated jointly by the patient and the provider, and are used for purposes beyond clinical care, including for research, operations, payments, quality control, and public health. The patient serves as the “data controller” with a reasonable say in which of their data are made available, to whom, and when. A “data processor” co-creates and adds data to the patient’s health record and accesses it when implicitly or explicitly authorized to do so. When patients cannot consent for lack of capacity, illiteracy, or circumstance, regulations should favor the patient’s best interest [85]. It might be useful to think about control in the context of a tiered hierarchy of permissions. The patient in most respects would be the final arbiter. Below the patient is a category of stakeholders with access to the data because they have been involved in its creation and to whom the patient has given implicit or explicit consent. Subordinate to these creators of data will be various other actors who can only gain access to the data with the patient’s permission or, if permitted by law, without [86].

In March 2018, the MoHFW invited public comment on a new bill it has proposed, the Digital Information Security in Health Care Act [87]. These evolving privacy and data protection laws in India must provide for deterrents to data misuse, where violations result in hefty fines, loss of access, and censure. On the other hand, an onerous, manual, consent-driven process would prevent patients from benefiting from advances in voice-activated services, machine learning, and artificial intelligence. Users of health data must therefore, above all, be expected to have a fiduciary responsibility toward the patient.

## Conclusion

In July 2018, the Government of India's NITI Aayog, the National Institute for Transforming India, published a blueprint for a "National Health Stack," embracing the principles of federated patient-centric data flows outlined in this paper [88,89]. The proposed “Health Stack” platform has the potential for revolutionizing medical care, research, and health care delivery in India. However, a simple, claims-driven model risks replicating the pitfalls of the US system [55]. It is also not likely to inspire provider or patient adoption.

For this proposed technological framework to meet its game-changing potential, the model will benefit from adhering to the following principles: (1) adopting a federated architecture, (2) prioritizing patient and population health needs over billing needs, (3) guaranteeing a patient’s right to her structured data, (4) allowing a plug and play model of highly customizable applications that can address varying context-specific needs, and that respond to market incentives for better user-interfaces, (5) mandating minimum data sets, (6) adopting privacy by design: automate audited and consented data flow, and finally (7) defaulting to safeguarding patients’ control over their data.

For patients, scientists and clinical providers to recognize, adopt and benefit from the vast potential of a secure, federated health information ecosystem, we propose a suite of initial applications whose benefits to society are palpable. For example, medication alerts, laboratory trends, schedulers and payment logs would prove highly useful to patients but would require interoperability among different sources of data, mandated or incentivized by the state. At a population level, disease surveillance data for modeling and forecasting outbreaks would be particularly useful to public health agencies. Standardized registries for trauma, cancer, rare diseases are desperately needed in India and can be built on the proposed framework. Aggregated and anonymized data sets accessed through an audited trail would help accelerate medical research, given the sheer volume of patient load in India.

However, such widespread adoption and data transfer between entities would necessitate buy-in from multiple stakeholders—through a combination of incentivization, legal mandate, budgetary allocation, and market demand for patient–provider, provider–provider, provider–payer, and payer–patient interactions [22]. There are no PHRs in India. It is precisely the greenfield nature of the digital health ecosystem in India that would allow a PHR-based, API–enabled network from the get-go, pre-empting the development of complex and incompatible silos of health data.

India must take advantage of its vibrant IT ecosystem and the widespread adoption of mobile technologies across its socio-economic strata. A light and robust API–enabled spine upon which both the public and private sector can be invited to build contextually appropriate, competing, substitutable, and incremental solutions will be the key to a forward-looking digital health ecosystem. The time for large centralized data warehouses and homogenous systems has passed.

There is much excitement globally about the power of big data, artificial intelligence and machine learning in reshaping medicine and health care delivery. However, the potential of these promising sciences can only be harnessed with reliable and timely data. A federated PHR in India will provide unprecedented amounts of health data; among them may lie answers to our well-being and happiness.

## Abbreviations

API: application programming interface
EHR: electronic health record
FHIR: Fast Healthcare Interoperability Resources
GDPR: General Data Protection Regulation
HIE: Health Information Exchange
HITECH: Information Technology for Economic and Clinical Health
ID: identification
IHIP: Integrated Health Information Platform
IT: information technology
KYC: know your customer
LOINC: Logical Observation Identifiers Names and Codes
MoHFW: The Ministry of Health and Family Welfare
NHPS: National Health Protection Scheme
PHR: personal health record
SNOMED-CT: Systematized Nomenclature of Medicine-Clinical Terms
